# *In vitro* Activity of Lefamulin Against the Common Respiratory Pathogens Isolated From Mainland China During 2017–2019

**DOI:** 10.3389/fmicb.2020.578824

**Published:** 2020-09-16

**Authors:** Shi Wu, Yonggui Zheng, Yan Guo, Dandan Yin, Demei Zhu, Fupin Hu

**Affiliations:** ^1^Institute of Antibiotics, Huashan Hospital, Fudan University, Shanghai, China; ^2^Key Laboratory of Clinical Pharmacology of Antibiotics, Ministry of Health, Shanghai, China

**Keywords:** lefamulin, antimicrobial susceptibility test, minimum inhibitory concentration, community-acquired bacterial pneumonia, *Mycoplasma pneumoniae*

## Abstract

**Purpose:**

Lefamulin is a novel antibiotic approved by the U.S. Food and Drug Administration in 2019 for the treatment of community-acquired bacterial pneumonia (CABP). In this study we evaluated the *in vitro* antimicrobial activity of lefamulin in order to better understand its antibiogram.

**Methods:**

The test strains were isolated from patients across China during the period from 2017 to 2019, including 634 strains of respiratory pathogens. The minimum inhibitory concentrations (MICs) of lefamulin and comparators were determined by broth microdilution method.

**Results:**

Lefamulin showed potent activity against *Streptococcus pneumoniae* and *Staphylococcus* evidenced by 100% inhibition at 0.25 mg/L, and favorable MIC_50/90_ (0.125/0.125 mg/L) against *S. pneumoniae* (penicillin MIC ≥ 2 mg/L), MIC_50/90_ (≤0.015/0.125 mg/L) against methicillin-resistant *S. aureus*, and MIC_50/90_ (≤0.015/0.06 mg/L) against methicillin-resistant *S. epidermidis*. Lefamulin also had good activity against *Streptococcus pyogenes* and *Streptococcus agalactia* (MIC_50/90_: ≤0.015/≤0.015 mg/L), β-lactamase-producing *Haemophilus influenzae* (MIC_50/90_: 0.5/1 mg/L), β-lactamase-negative *H. influenzae* (MIC_50/90_: 1/1 mg/L), *Moraxella catarrhalis* (MIC_50/90_: 0.25/0.25 mg/L), and *Mycoplasma pneumoniae* (MIC_50/90_: 0.03/0.03 mg/L) regardless of resistance to azithromycin. Lefamulin was generally more active than the comparators against the test strains.

**Conclusion:**

In summary, lefamulin has good and broad-spectrum coverage of respiratory pathogens (methicillin-sensitive and -resistant *Staphylococcus*, *S. pneumoniae*, β-hemolytic *Streptococcus*, *H. influenzae*, *M. catarrhalis* and *M. pneumoniae*). *In vitro* activity supports the use of lefamulin in the treatment of CABP in China.

## Introduction

Pleuromutilin is a natural antimicrobial substance first found in 1950s. It can be obtained from *Clitopilus scyphoides*, *Clitopilus passeckerianus*, or other *Clitopilus* species in basidiomycota. Lefamulin is the first-in-class semi-synthetic pleuromutilin antibiotic for systemic use. Its molecular formula is C_28_H_45_NO_5_S (molecular weight 567.79 g). Lefamulin inhibits bacterial protein synthesis by binding to “A” and “P” sites of the peptidyl transferase center (PTC) of the 23s rRNA of the 50S ribosomal subunit of bacterial cell. The binding is through the mutilin core and C-14 side chain in the forms of hydrogen bonds, hydrophobic interactions, and conformational change to prevent correct orientation of tRNA’s 3’-CCA ends for peptide transfer (Veve and Wagner, 2018; Rodvold, 2019). The resistance to lefamulin may be related to the mutations in *rplC* gene and *cfr* gene of *Staphylococcus aureus*, Vga (AV) coded by the transposon Tn5406 and *vga(A)* carried by plasmids (encoding ABC transporter) (Mendes et al., 2019; Rodvold, 2019). So far, it is known that lefamulin has no cross resistance to the antimicrobial agents in clinical use.

Studies have shown that lefamulin has good coverage of the pathogens of community-acquired respiratory tract infections, including antibiotic-resistant strains, such as penicillin-resistant *Streptococcus pneumoniae* (PRSP), macrolide-resistant *Mycoplasma pneumoniae*, and methicillin-resistant *S. aureus* (MRSA) (Veve and Wagner, 2018; Rodvold, 2019). In August 2019, lefamulin was approved by the U.S. Food and Drug Administration (FDA) for the treatment of community-acquired bacterial pneumonia (CABP) patients based on its good pharmacodynamic results, pharmacokinetic, and safety profiles in clinical trials.

The antibacterial spectrum and activity of lefamulin have been studied in the United States and Europe (Paukner et al., 2013, 2019), but it is not clear about its antimicrobial activity against the clinical isolates in China. For better understanding the antimicrobial activity of lefamulin against the common respiratory pathogens recently isolated in China, we studied the *in vitro* activity of lefamulin against a broad range of respiratory pathogens.

## Materials and Methods

A total of 634 non-duplicate strains of respiratory pathogens were tested, including 580 strains of bacteria and 54 strains of *Mycoplasma pneumoniae*. These strains were isolated from 29 hospitals across China, representing 23 provinces and municipalities, during the period from October 2017 to July 2019. Specifically, the test strains included *S. aureus* (*n* = 121), *S. epidermidis* (*n* = 30), β-lactamase-producing *Haemophilus influenzae* (*n* = 48), β-lactamase-negative *H. influenzae* (*n* = 48), *Haemophilus parainfluenzae* (*n* = 10), *Moraxella catarrhalis* (*n* = 54), *S. pneumoniae* (*n* = 172), *Streptococcus pyogenes* (*n* = 30), and *Streptococcus agalactiae* (*n* = 13). All the strains were re-identified before susceptibility testing. Species identification was confirmed by MALDI-TOF/MS system (bioMérieux, France), and antimicrobial susceptibility testing were controlled with reference strains *S. aureus* ATCC29213, *S. pneumoniae* ATCC49619, *H. influenzae* ATCC49247, and *M. pneumoniae* ATCC 29342.

The minimum inhibitory concentrations (MICs) of lefamulin and the comparators were determined by broth microdilution method according to the Clinical and Laboratory Standards Institute (CLSI) (2018) M07-11th Edition (CLSI, 2018). The MICs against *M. pneumoniae* were measured according to the methods for antimicrobial susceptibility testing for human mycoplasmas described in CLSI document M43-A (2011) (CLSI, 2011). The antimicrobial comparators included tigecycline, moxifloxacin, linezolid, penicillin, ampicillin, oxacillin, ceftriaxone, levofloxacin, vancomycin, trimethoprim-sulfamethoxazole, erythromycin, and azithromycin. The concentrations of the test antimicrobial agents ranged from 32 mg/L to 0.015 mg/L.

WHONET 5.6 software and the breakpoints of CLSI M100-29^*th*^ Edition (CLSI, 2019) were used to interpret and analyze the results of antimicrobial susceptibility test. Lefamulin and tigecycline were analyzed according to the breakpoints recommended by FDA^1^. The breakpoints of lefamulin was ≤0.25 mg/L active against methicillin-sensitive *S. aureus*, ≤0.5 mg/L against *S. pneumoniae*, and ≤2 mg/L against *H. influenzae*. The breakpoint of tigecycline was ≤0.5 mg/L active against *S. aureus*, and ≤0.25 mg/L against *H. influenzae*.

### Ethics Statement

The study protocol was approved by the Ethics Committee of Huashan Hospital, Fudan University (Number: 2019-319).

## Results

**FIGURE 1:**
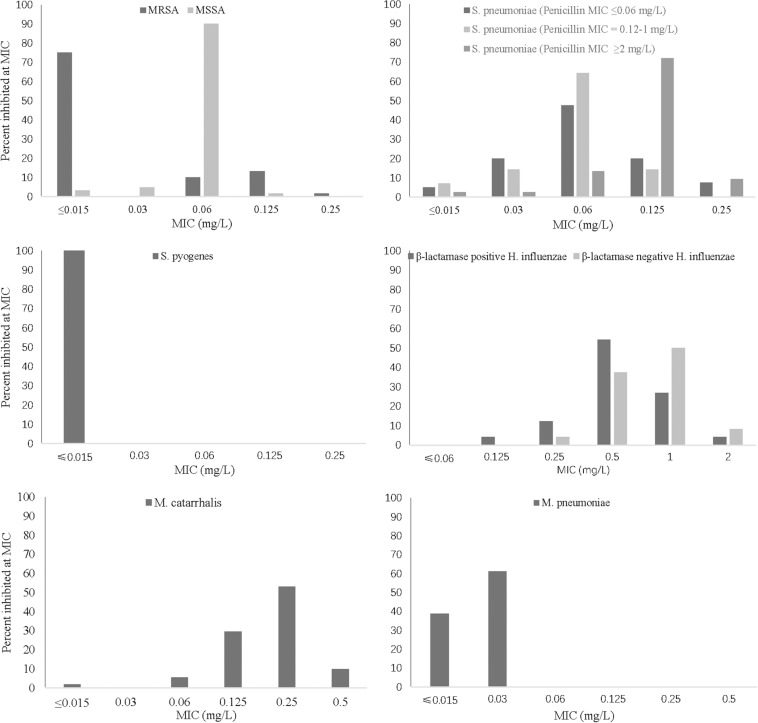
MIC frequency distribution of lefamulin against respiratory pathogens. MRSA (*n* = 60), MSSA (*n* = 61), *S. pneumoniae* strains (penicillin MIC ≤ 0.06 mg/L, *n* = 40; penicillin MIC = 0.12–1 mg/L, *n* = 40; penicillin MIC ≥ 2 mg/L, *n* = 118), *S. pyogenes* (*n* = 30), *H. influenzae* (β-lactamase-positive strains, *n* = 48; β-lactamase-negative strains, *n* = 48), *M. catarrhalis* (*n* = 54), and *M. pneumoniae* (*n* = 54). MIC, minimum inhibitory concentration; MRSA, methicillin-resistant *S. aureus*; MSSA, methicillin-susceptible *S. aureus.*

Lefamulin at 0.25 mg/L inhibited the growth of all *Staphylococcus* strains (Table 1 and Figure 1). The MIC_90_ value of lefamulin was 0.125 mg/L against MRSA, 0.06 mg/L against methicillin-resistant *S. epidermidis* (MRSE), 0.06 mg/L against methicillin-sensitive *S. aureus* (MSSA), and 0.03 mg/L against methicillin-sensitive *S. epidermidis* (MSSE). Lefamulin displayed MIC values ranging from ≤0.015 mg/L to 0.25 mg/L (MIC_90_: ≤0.25 mg/L) against 172 strains of *S. pneumoniae*, including penicillin-susceptible (PSSP) strains (penicillin MIC ≤0.06 mg/L), penicillin-intermediate (PISP) strains (penicillin MIC: 0.125 mg/L–1 mg/L), and penicillin-resistant (PRSP) strains (penicillin MIC ≥ 2 mg/L). Lefamulin inhibited the growth of all PSSP strains at ≤0.015 mg/L and all PISP and PRSP strains at 0.25 mg/L. The MIC_50/90_ values of lefamulin were ≤0.015/≤0.015 mg/L against *S. pyogenes* and ≤0.015/0.06 mg/L against *S. agalactiae*. Lefamulin inhibited the growth of all the *S. pyogenes* and *S. agalactiae* strains at 0.06 mg/L (Table 2 and Figure 1).

**TABLE 1:** *In vitro* activity of lefamulin and comparators against *Staphylococcus.*

Organism (No. of strains)	Antimicrobial agent	MIC (mg/L)			R%	S%
		MIC Range	MIC_50_	MIC_90_		
MRSA (*n* = 60)	Lefamulin	≤0.015−0.25	≤0.015	0.125	–	–
	Oxacillin	32 – >32	>32	>32	100	0
	Levofloxacin	0.125–>32	8	>32	53.3	45
	Moxifloxacin	0.03–16	1	8	48.3	48.3
	Erythromycin	0.25–>32	>32	>32	85.0	11.7
	Azithromycin	0.5–>32	>32	>32	85.0	15.0
	Vancomycin	0.5–2	1	1	0	100
	Linezolid	0.5–4	2	2	0	100
	Tigecycline	0.06–0.5	0.125	0.25	–	100
MSSA (*n* = 61)	Lefamulin	≤0.015–0.125	0.06	0.06	**–**	100
	Oxacillin	0.25–2	1	2	0	100
	Levofloxacin	0.125–8	0.25	0.5	3.3	96.7
	Moxifloxacin	≤0.015–2	0.06	0.25	3.3	96.7
	Erythromycin	0.25–>32	>32	>32	55.7	44.3
	Azithromycin	0.5–>32	>32	>32	55.7	44.3
	Vancomycin	0.5–1	1	1	0	100
	Linezolid	1–4	2	4	0	100
	Tigecycline	0.06–0.25	0.125	0.25	**–**	100
MRSE (*n* = 15)	Lefamulin	≤0.015–0.125	≤0.015	0.06	**–**	**–**
	Oxacillin	1–>32	4	32	100	0
	Levofloxacin	0.125–>32	8	>32	73.3	20
	Moxifloxacin	0.03–32	2	32	53.3	20
	Erythromycin	0.25–>32	>32	>32	86.7	13.3
	Azithromycin	0.25–>32	>32	>32	80.0	20.0
	Vancomycin	1–2	1	2	0	100
	Linezolid	0.25–1	1	1	0	100
	Tigecycline	0.06–0.25	0.06	0.25	**–**	**–**
MSSE (*n* = 15)	Lefamulin	≤0.015–0.06	≤0.015	0.03	**–**	**–**
	Oxacillin	0.06–0.125	0.125	0.125	0	100
	Levofloxacin	0.125–4	0.25	4	13.3	80
	Moxifloxacin	0.03–1	0.06	0.5	0	93.3
	Erythromycin	0.125–>32	32	>32	60.0	40.0
	Azithromycin	0.125–>32	32	>32	60.0	40.0
	Vancomycin	1–2	1	2	0	100
	Linezolid	0.5–2	1	1	0	100
	Tigecycline	0.06–0.25	0.125	0.25	**–**	–

*MIC, minimum inhibitory concentration; MIC_50_, MIC for inhibiting 50% of the isolates; MIC_90_, MIC for inhibiting 90% of the isolates; R, resistant; S, susceptible; MRSA, methicillin-resistant *S. aureus*; NA, not available; MSSA, methicillin-susceptible *S. aureus*; MRSE, methicillin-resistant *S. epidermidis*; MSSE, methicillin-susceptible *S. epidermidis.**

**TABLE 2:** *In vitro* activity of lefamulin and comparators against *Streptococcus* species.

Organism (No. of strains)	Antimicrobial agent	MIC (mg/L)			R%	S%
		MIC Range	MIC_50_	MIC_90_		
*Streptococcus pneumoniae* (penicillin MIC ≤ 0.06 mg/L) (*n* = 40)	Lefamulin	≤0.015–≤0.015	≤0.015	≤0.015	–	100
	Ceftriaxone	≤0.015–0.03	≤0.015	≤0.015	0	100
	Penicillin	≤0.015–≤0.015	≤0.015	≤0.015	0	100
	Levofloxacin	0.125–0.25	0.25	2	3.3	97.5
	Moxifloxacin	0.03–0.25	0.06	0.125	0	97.5
	Erythromycin	0.03–>32	>32	>32	97.5	2.5
	Azithromycin	0.06–>32	>32	>32	97.5	2.5
	Vancomycin	≤0.015–0.5	0.25	0.5	0	100
	Linezolid	0.125–1	1	1	0	100
*Streptococcus pneumoniae* (penicillin MIC = 0.12–1 mg/L) (*n* = 40)	Lefamulin	0.03–0.25	0.125	0.25	–	100
	Ceftriaxone	0.03–1	0.125	0.5	0	100
	Penicillin	0.125–1	0.5	1	–	–
	Levofloxacin	0.5–2	1	1	0	100
	Moxifloxacin	0.125–0.25	0.125	0.25	0	100
	Erythromycin	2–>32	>32	>32	100	0
	Azithromycin	2–>32	>32	>32	100	0
	Vancomycin	0.06–0.5	0.25	0.5	–	100
	Linezolid	0.25–1	1	1	–	100
*Streptococcus pneumoniae* (penicillin MIC ≥ 2 mg/L) (*n* = 118)	Lefamulin	≤0.015–0.25	0.125	0.125	–	100
	Ceftriaxone	0.5–>32	2	4	45.8	47.5
	Penicillin	2–32	8	8	100	0
	Levofloxacin	0.125–32	1	1	1.7	98.3
	Moxifloxacin	0.06–8	0.125	0.25	0.8	98.3
	Erythromycin	2–>32	>32	>32	100	0
	Azithromycin	2–>32	>32	>32	100	0
	Vancomycin	0.125–1	0.25	0.5	–	100
	Linezolid	0.25–2	1	1	–	100
*Streptococcus pyogenes* (*n* = 30)	Lefamulin	≤0.015–≤0.015	≤0.015	≤0.015	–	–
	Ceftriaxone	≤0.015–0.03	≤0.015	≤0.015	0	100
	Penicillin	≤0.015–≤0.015	≤0.015	≤0.015	0	100
	Levofloxacin	0.125–0.25	0.25	2	3.3	96.7
	Moxifloxacin	0.03–0.25	0.06	0.125	–	–
	Erythromycin	0.03–>32	>32	>32	93.3	6.7
	Azithromycin	0.06–>32	>32	>32	93.3	6.7
	Vancomycin	0.25–0.5	0.25	0.5	0	100
	Linezolid	0.5–2	1	1	0	100
*Streptococcus agalactiae* (*n* = 13)	Lefamulin	≤0.015–0.03	≤0.015	0.03	–	–
	Ceftriaxone	≤0.015–0.06	≤0.015	0.06	0	100
	Penicillin	0.06–0.125	0.06	0.06	0	100
	Levofloxacin	0.5–1	1	1	0	100
	Moxifloxacin	0.125–0.25	0.125	0.25	–	–
	Erythromycin	0.06–>32	2	>32	69.2	30.8
	Azithromycin	0.06 –>32	16	>32	69.2	30.8
	Vancomycin	0.5–0.5	0.5	0.5	–	100
	Linezolid	1–2	1	2	–	100

*MIC, minimum inhibitory concentration; MIC_50_, MIC for inhibiting 50% of the isolates; MIC_90_, MIC for inhibiting 90% of the isolates; R, resistant; S, susceptible.*

The MIC_50/90_ values of lefamulin ≤ 1/ ≤ 1 mg/L against *H. influenzae* and *H. parainfluenzae*, regardless of β-lactamase production. Lefamulin inhibited the growth of all the *Haemophilus* strains at 2 mg/L (Table 3 and Figure 1). Lefamulin showed MIC_50/90_ of 0.25/0.25mg/L against *M. catarrhalis*. Lefamulin inhibited the growth of all *M. catarrhalis* strains at 0.5 mg/L (Table 3 and Figure 1).

**TABLE 3:** *In vitro* activity of lefamulin and comparators against *Haemophilus influenzae* and *Moraxella catarrhalis.*

Organism (No. of strains)	Antimicrobial agent	MIC (mg/L)			R%	S%
		MIC Range	MIC_50_	MIC_90_		
*Haemophilus influenzae* (β-lactamase positive) (*n* = 48)	Lefamulin	0.125–2	0.5	1	–	100
	Ceftriaxone	≤0.015–1	0.03	0.125	–	100
	Ampicillin	8–>32	>32	>32	100	0
	Levofloxacin	≤0.015–1	≤0.015	0.5	–	100
	Moxifloxacin	≤0.015–1	≤0.015	0.5	–	100
	Erythromycin	4–>32	>32	>32	–	NA
	Azithromycin	1–>32	>32	>32	–	27.1
	Tigecycline	0.06–0.25	0.125	0.25	–	100
	Trimethoprim-sulfamethoxazole	0.06/1.14–32/608	8/152	16/304	77.1	16.7
*Haemophilus influenzae* (β-lactamase negative) (*n* = 48)	Lefamulin	0.25–2	1	1	–	100
	Ceftriaxone	≤0.015–0.25	≤0.015	0.06	–	100
	Ampicillin	≤0.015–1	0.5	1	0	100
	Levofloxacin	≤0.015–1	≤0.015	0.5	–	100
	Moxifloxacin	≤0.015–1	≤0.015	0.5	–	100
	Erythromycin	2–>32	8	8	–	NA
	Azithromycin	0.5–>32	2	2	–	97.9
	Tigecycline	0.125–0.25	0.25	0.25	–	100
	Trimethoprim-sulfamethoxazole	0.03/0.57–16/304	4/76	16/304	56.2	37.5
*Haemophilus parainfluenzae* (*n* = 10)	Lefamulin	0.015–2	0.5	1	–	–
	Ceftriaxone	0.015–0.25	0.03	0.125	0	100
	Ampicillin	≤0.015–8	0.125	4	30.0	70.0
	Levofloxacin	0.03–8	0.125	4	0	80.0
	Moxifloxacin	0.125–16	0.25	4	0	60.0
	Erythromycin	2 –>32	2	8	–	–
	Azithromycin	0.25–16	1	2	0	90
	Tigecycline	0.125–1	0.5	0.5	–	–
	Trimethoprim-sulfamethoxazole	0.015/0.285–16/304	0.125/2.375	2/38	10	70
*Moraxella catarrhalis* (*n* = 54)	Lefamulin	≤0.015–0.5	0.25	0.25	–	–
	Ceftriaxone	≤0.015–2	0.5	1	0	100
	Ampicillin	≤0.015–>32	1	4	–	–
	Levofloxacin	≤0.015–1	0.06	0.06	0	100
	Moxifloxacin	≤0.015–0.5	0.06	0.06	–	–
	Erythromycin	0.125–>32	1	>32	–	–
	Azithromycin	0.03–>32	0.25	>32	0	66.7
	Tigecycline	0.03–2	0.06	0.125	–	–
	Trimethoprim-sulfamethoxazole	0.03/0.57 –>32/608	0.5/9.5	4/76	11.1	64.8

*MIC, minimum inhibitory concentration; MIC_50_, MIC for inhibiting 50% of the isolates; MIC_90_, MIC for inhibiting 90% of the isolates; R, resistant; S, susceptible; NA, not available.*

Lefamulin inhibited the growth of all *M. pneumoniae* strains at 0.03 mg/L. The MIC ranged from ≤0.015 to 0.03 mg/L (MIC_50/90_: 0.03/0.03 mg/L). Its activity was comparable to moxifloxacin and significantly superior to erythromycin and azithromycin (Table 4 and Figure 1).

**TABLE 4:** *In vitro* activity of lefamulin and comparators against *M. pneumoniae.*

Organism (no. of strains)	Antimicrobial agent	MIC (mg/L)			R%	S%
		MIC Range	MIC_50_	MIC_90_		
*Mycoplasma pneumoniae* (*n* = 54)	Lefamulin	≤0.015 –0.03	0.03	0.03	–	–
	Erythromycin	≤0.015–>32	32	>32	94.4	5.6
	Azithromycin	≤0.015–32	8	16	94.4	5.6
	Moxifloxacin	0.06–0.125	0.06	0.125	–	100

*MIC, minimum inhibitory concentration; MIC_50_, MIC for inhibiting 50% of the isolates; MIC_90_, MIC for inhibiting 90% of the isolates; R, resistant; S, susceptible; NA, not available.*

## Discussion

In the present study, lefamulin displayed excellent antimicrobial activity against all the respiratory pathogens, including MRSA, MSSA, MRSE, MSSE, *S. pneumoniae*, β-hemolytic *Streptococcus*, *Haemophilus*, *M. catarrhalis*, and *M. pneumoniae*. Our results are consistent with the reports of Susanne Paukner et al. on the antimicrobial activity of lefamulin against 1,473 and 2,661 strains of *S. pneumoniae*, 3,923 and 2,919 strains of *S. aureus* in the SENTRY Antimicrobial Surveillance Program 2010 and 2015–2016 (Paukner et al., 2013, 2019). The MIC_90_ of lefamulin was 0.25 mg/L and 0.12 mg/L against *S. pneumoniae*, regardless of resistance to penicillin, ceftriaxone and/or levofloxacin. The MIC_50/90_ was 0.12/0.12 mg/L against MRSA and MSSA. They also reported that the MIC value of lefamulin was 2–>16 mg/L against two MRSA isolates and 5 MSSA isolates in 2010, whereas the MIC value of lefamulin against 11 *S. aureus* isolates in 2015–2016 was higher than its epidemiological cutoff value. However, all the *Staphylococcus* strains tested in the present study were sensitive to lefamulin. All the *Staphylococcus* strains were also susceptible to tigecycline, vancomycin, and linezolid. However, lefamulin inhibited the growth of all *Staphylococcus* strains at concentration of ≤0.25 mg/L, which is far lower than the concentration of 1–2, 1–4, and 0.25–0.5 mg/L required by the above three comparators for 100% inhibition of bacterial growth. Lefamulin was also superior to quinolones (only inhibited 80–96.7% of the strains) in this respect.

Lefamulin also displayed high antimicrobial activity against *Haemophilus* and *M. catarrhalis*. Lefamulin was comparable to ceftriaxone in activity against *S. pneumoniae* strains (PSSP, PISP) and β-hemolytic *Streptococcus*, but better than ceftriaxone against PRSP, better than penicillin against PISP and PRSP, and similar to penicillin against β-hemolytic *Streptococcus*. Lefamulin had similar activity as moxifloxacin, vancomycin, and linezolid against *Streptococcus*. It inhibited the growth of all *Streptococcus* species at 0.125 mg/L, which was lower than the above mentioned three agents. Lefamulin was significantly better than erythromycin and azithromycin in the activity against *S. pneumoniae* and β-hemolytic *Streptococcus*.

In this study, lefamulin also had good antimicrobial effect on the gram-negative bacilli commonly found in CABP. Lefamulin was similar to ceftriaxone, tigecycline, levofloxacin, and moxifloxacin, and better than ampicillin, azithromycin, and trimethoprim-sulfamethoxazole in the activity against β-lactamase-producing *H. influenzae* and *M. catarrhalis*. As for the β-lactamase-negative strains, lefamulin provided significantly better activity than azithromycin. Lefamulin was comparable to tigecycline, ceftriaxone, and levofloxacin, and significantly superior to azithromycin and trimethoprim-sulfamethoxazole in the activity against *M. catarrhalis*. These results are consistent with those reports from other countries (Paukner et al., 2013, 2019).

It has been reported that the *M. pneumonia*e strains isolated from China are highly resistant to macrolides. Our results also confirmed the previous reports. About 94.4% of the 54 *M. pneumoniae* strains were resistant to erythromycin and azithromycin in this study. However, lefamulin still showed MIC range from ≤0.015 to 0.03 mg/L, which was not affected by resistance to macrolides. This MIC range is consistent with that from other countries (MIC_90_: 0.002 mg/L) (Waites et al., 2017).

Lefamulin is the first semi-synthetic pleuromutilin antimicrobial agent approved for the treatment of CABP patients. Clinical trials have proved the excellent therapeutic effect of lefamulin. The MIC_90_ value of lefamulin was 0.5 μg/mL against the 50 strains of *S. pneumoniae* isolated from the patients in phase III clinical trial LEAP 1 (File et al., 2019) and 0.25 μg/mL against the 123 strains of *S. pneumoniae* isolated from the patients in clinical trial LEAP 2 (Alexander et al., 2019). The MIC_90_ against *S. aureus* isolates (10 and 13 strains) was 0.12–0.25 μg/mL. The post-treatment bacterial clearance rate was up to 100%. Research results at home and abroad have shown that lefamulin had similar antimicrobial activity against *S. epidermidis* and *S. aureus* (Paukner et al., 2013, 2019).

The above results support the excellent antimicrobial activity of lefamulin against CABP pathogens, especially antibiotic-resistant pathogens, such as PRSP, macrolide-resistant *M. pneumoniae* and MRSA. The major parameter driving efficacy for both *S. aureus* and *S. pneumoniae* is the 24h area under the drug concentration–time curve (AUC) over the MIC (24 h AUC/MIC). Lefamulin achieves rapid and predictable penetration into human tissues, with a mean 5.7-fold higher concentration in the pulmonary epithelial lining fluid compared with plasma. Percent probabilities of attaining the median AUCELF/MIC ratio targets associated with a 1-log10 CFU reduction from baseline by MIC were 97.0% at a MIC of 0.5 μg/mL for *S. pneumoniae* and 99.4% at a MIC of 0.25 μg/mL for *S. aureus* (Falcó et al., 2020). The unique mechanism of action, lack of cross resistance, good and broad coverage of respiratory pathogens regardless of resistance to other antimicrobial agent (Abbas et al., 2017; Lee and Jacobs, 2019) will surely make lefamulin a promising alternative treatment option in Chinese patients with CABP, especially those caused by PRSP, MRSA, or macrolide-resistant *M. pneumoniae*.

## Data Availability Statement

The raw data supporting the conclusions of this article will be made available by the authors, without undue reservation.

## Ethics Statement

The study protocol was approved by the Ethics Committee of Huashan Hospital, Fudan University (Number: 2019-319).

## Author Contributions

DZ and FH designed the study. SW, YZ, YG, and DY performed the experimental work. SW and YZ collected the data. FH analyzed the data. All authors read and approved the final manuscript, contributed to the article, and approved the submitted version.

## Conflict of Interest

The authors declare that the research was conducted in the absence of any commercial or financial relationships that could be construed as a potential conflict of interest.

## Footnotes

1 www.fda.gov/drugs/development-resources/antibacterial-susceptibility-test-interpretive-criteria
